# A Comparative Study on Carbohydrate Estimation: GoCARB vs. Dietitians

**DOI:** 10.3390/nu10060741

**Published:** 2018-06-07

**Authors:** Maria F. Vasiloglou, Stavroula Mougiakakou, Emilie Aubry, Anika Bokelmann, Rita Fricker, Filomena Gomes, Cathrin Guntermann, Alexa Meyer, Diana Studerus, Zeno Stanga

**Affiliations:** 1 Diabetes Technology Research Group, ARTORG Center for Biomedical Engineering Research, University of Bern, Murtenstrasse 50, 3008 Bern, Switzerland; maria.vasiloglou@artorg.unibe.ch; 2 Division of Diabetes, Endocrinology, Metabolism and Clinical Nutrition, Bern University Hospital “Inselspital”, 3010 Bern, Switzerland; Emilie.Aubry@insel.ch (E.A.); zeno.stanga@insel.ch (Z.S.); 3 Diabetes Centre for Children and Adolescents, Children’s and Youth Hospital “Auf Der Bult”, Janusz-Korczak-Allee 12, 30173 Hannover, Germany; a.bokelmann@bernward-khs.de (A.B.); guntermann@hka.de (C.G.); 4 Gewerblich-Industrielle Berufsschule Bern (GIBB), Lorrainestrasse 1, 3000 Bern, Switzerland; rita.fricker@bluewin.ch; 5 Medical University Department, Kantonsspital Aarau, Tellstrasse 25, 5001 Aarau, Switzerland; filomenisabel@hotmail.com; 6 Faculty of Life Sciences, University of Vienna, Althanstrasse 14, 1090 Vienna, Austria; alexa.leonie.meyer@univie.ac.at; 7 Food on Record, Freie Strasse 59, 4001 Basel, Switzerland; diana@foodonrecord.com

**Keywords:** type 1 diabetes, dietitian, visual estimation, carbohydrate counting, computer vision, artificial intelligence, smartphone

## Abstract

GoCARB is a computer vision-based smartphone system designed for individuals with Type 1 Diabetes to estimate plated meals’ carbohydrate (CHO) content. We aimed to compare the accuracy of GoCARB in estimating CHO with the estimations of six experienced dietitians. GoCARB was used to estimate the CHO content of 54 Central European plated meals, with each of them containing three different weighed food items. Ground truth was calculated using the USDA food composition database. Dietitians were asked to visually estimate the CHO content based on meal photographs. GoCARB and dietitians achieved comparable accuracies. The mean absolute error of the dietitians was 14.9 (SD 10.12) g of CHO versus 14.8 (SD 9.73) g of CHO for the GoCARB (*p* = 0.93). No differences were found between the estimations of dietitians and GoCARB, regardless the meal size. The larger the size of the meal, the greater were the estimation errors made by both. Moreover, the higher the CHO content of a food category was, the more challenging its accurate estimation. GoCARB had difficulty in estimating rice, pasta, potatoes, and mashed potatoes, while dietitians had problems with pasta, chips, rice, and polenta. GoCARB may offer diabetic patients the option of an easy, accurate, and almost real-time estimation of the CHO content of plated meals, and thus enhance diabetes self-management.

## 1. Introduction

The worldwide incidence of type 1 diabetes (T1D) is increasing by 3% a year [1]. In high income countries, more than half a million children and 7–12% of the general population are affected by the disease. In Europe, there are ca. 140,000 cases in all and ~21,600 new cases per year [1].

T1D treatment relies on maintaining good glycaemic control, and this is a key component of diabetes self-management. The consumed carbohydrates (CHO) are a major determinant of postprandial blood glucose and the current guidelines recommend that the amount of CHO in the meal should be taken into account for prandial insulin calculation [2]. CHO counting is an approach to planning meals, and it is focused on the estimation of the macronutrient content that mostly influences postprandial glycaemic response. In individuals with T1D, this is a more effective tool for improving hemoglobin A1c (HbA1c) concentrations than any sort of dietary education [3]. A systematic review and meta-analysis estimated that there is a 0.64% reduction in HbA1c in patients who undertook CHO counting versus control [4].

In general, individuals with T1D, even trained and experienced, are not accurate in CHO counting [5,6,7,8]. In a cross-sectional study, T1D adults (*n* = 50) underestimated 63% of the meals analysed, with a mean error of 20% of total CHO content per meal [5]. Moreover, of 61 patients with T1D, 82% overestimated CHO content by 40%, [6]. Other studies found that high CHO foods were underestimated not only by children, young adults with T1D and their parents, but also by health professionals [7], whereas adolescents with T1D tended to overestimate them [8]. Studies have shown that an error of ±10 g in CHO content estimation maintains postprandial control [9]. On the other hand, an error in the order of 20 g, CHO significantly impacts postprandial glycaemia, since it exceeds the limits covered by a single mealtime insulin dose, resulting in hypoglycaemia or hyperglycemia between 2–3 h after meal consumption [10].

With the advancement, usage and adoption of smartphone technology and the constant growth of the overall Mobile Heath (mHealth) ecosystem [11], opportunities for improved diabetes control have arisen. However, most of the existing apps often rely on mostly manual procedures, in which the user has to insert food names, and in some cases, to also add portion size (e.g., in cups or to select between small, medium, and large portionsize). Recent advances in hardware and software have enabled the implementation of applications (Apps) with intelligent new features using machine learning and computer vision algorithms. Thus, novel approaches for nutrient estimations based on food image [12,13,14,15] or video [13] analysis were introduced. Such approaches use algorithms to analyse the acquired food image or video, in order to automatically identify, label, segment, and quantify food items, in terms of volume [14], calories [13,16], and grams of macronutrients [17].

While there have been several attempts on diet assessment using machine learning and computer vision [12,13,15,18,19], only GoCARB is designed to support individuals with T1D and is clinically validated with respect to its impact on glucose control. GoCARB is a novel smartphone-based approach for CHO counting, employing computer vision, machine learning, and smartphone technologies [17]. The app has been developed by the Diabetes Technology Research (DTR), ARTORG Research Center for Biomedical Engineering of the University of Bern (Switzerland) [20,21]. The system was designed to support CHO estimation of plated meals for individuals with T1D living in German speaking countries. In a preclinical study, the accuracy of GoCARB was compared with CHO self-estimations of trained adults with T1D and it provided more accurate results [20]. A pilot prospective randomised crossover study (*n* = 20) assessed the effect of GoCARB use on postprandial glucose and overall glucose control, and demonstrated an improvement in the proportion of hyperglycaemic time and glucose variability [21].

The main objective of the present study is to investigate the differences in CHO estimation, by comparing the results that were obtained by visual estimation by (i) professional experienced dietitians, and (ii) GoCARB, with (iii) the actual weight of the meals provided (reference method). To the best of our knowledge, this is the first study that compares the accuracy of dietitians’ estimations, mobile technology, and the actual weight of meals.

## 2. Materials and Methods

GoCARB is designed to provide CHO estimations to individuals with T1D. A credit card-sized reference card (8.5 × 5.5 cm) is placed next to the dish and two images of the plated meal are acquired by the user using an Android smartphone in which GoCARB is installed. The images are taken from two different viewing angles. Once the image acquisition process is completed, a number of computational steps permit the recognition of the various food items and the meal’s volume estimation. By knowing the food type and the respective volume, and by using the USDA food composition database, the CHO content is estimated [17,20,22]. The procedure followed for GoCARB is described in detail elsewhere [20].

### 2.1. Study Design

**Figure 1:**
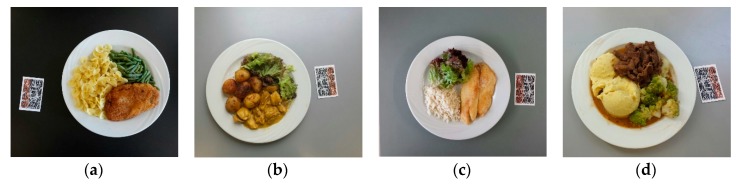
Sample images sent to dietitians.

Fifty-four dishes were ordered from the Bern University Hospital (“Inselspital”) restaurant over a period of two months (July, August 2014). The dishes corresponded to standard Central European meals as served by the restaurant to employees and visitors—examples are shown in Figure 1a–d and constitutes a subset of the dishes used in a previous study [20]. For each of the 54 meals, three different sizes were shown (small, medium, and large), with each plate containing three different food items: one high in protein (e.g., meat), one high in CHO (e.g., pasta) and one vegetable component (e.g., zucchini), and the items did not overlap. Fifteen food categories were considered; breaded meat (e.g., schnitzel), pasta, rice, potatoes, mashed potatoes, polenta, green beans, meat, carrots, salad (i.e., leafy vegetables), chips (French fries), noodles, zucchini, broccoli, and fish.

GoCARB was used to estimate the meals’ CHO content. GoCARB needs two photos that were acquired by different shooting angles to estimate the CHO content (1 GoCARB usage = 2 images). For each of the 54 dishes, GoCARB was used one to three times (1× for 13 meals; 2× for 25 meals; 3× for the rest 16 meals). Thus, two to six photos for each meal were taken resulting in a total of 111 GoCARB estimations and 222 meal images. Then, each food item was weighed using household scales (Kenwood, model AT850B) and ground truth (GTR) was estimated using the meals’ exact food items, as it appears in the USDA National Nutrient Database for Standard Reference.

**Figure 2:**
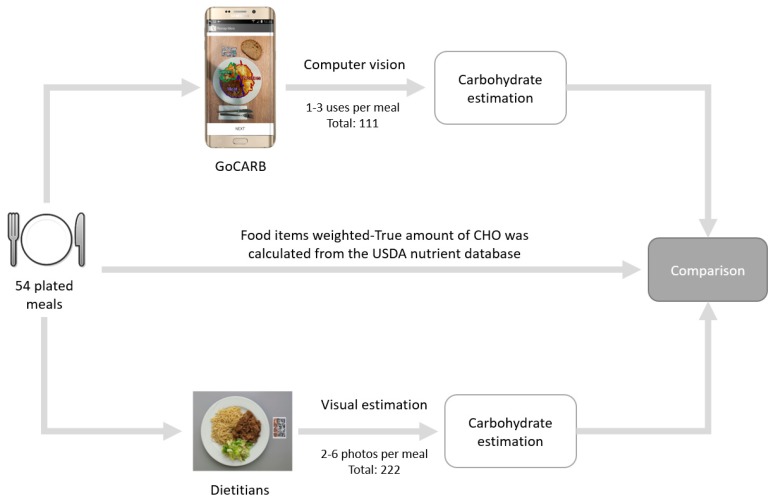
Outline of the procedure including dietitians and GoCARB.

The same 222 images were sent to a pool of dietitians in November 2016. Each dietitian was asked to calculate the corresponding CHO content of each image. Their answers were received by June 2017. The dietitians were advised to use the Diabetic Exchange List that was designed by a committee of the American Diabetes Association and the American Dietetic Association [23], but the final decision to use the proposed list was made by the dietitians. They were blinded to the actual weight of the meals, their CHO content, and the corresponding food items. An overview of the methodological approach that was used for GoCARB and the dietitians is presented in Figure 2.

### 2.2. Participants

Twelve dietitians from German speaking countries (i.e., Germany, Switzerland, and Austria) were personally contacted and informed about the study’s nature. Eligibility criteria required individuals to have received a BSc degree in Nutrition and Dietetics, be German speaking, and also to have had at least five years’ experience with diabetic patients and in CHO counting as professional dietitians. Six of them (four from Switzerland, one from Austria and one from Germany) replied positively and agreed to participate in the study (response rate 50%). An overview of the participants’ characteristics is provided in Table 1.

### 2.3. Data Analysis

Data were analysed using RStudio (Version 1.0.153—© 2009–2017 RStudio, Inc., Boston, MA, USA). Dietitians’ CHO estimations, as well as differences between visual estimation and the weighing method were described as mean ± standard deviation (SD). Differences between CHO estimation of the two methods were calculated as actual counts and absolute errors using the equation: difference = visual estimation minus weight. Absolute values were calculated in order to evaluate the accuracy of the visual estimation, while actual values were used to calculate over- and under-estimations by the dietitians and GoCARB. Since our data was not normally distributed according to the Shapiro-Wilk test (*p* < 0.05), we performed non-parametric analyses. Spearman’s correlation coefficient and Wilcoxon’s signed-rank test were used for the comparison of CHO content calculated by the dietitians’ visual estimation, GoCARB estimation, and weighing methods. The interrater reliability was assessed by using the intraclass correlation coefficients (ICCs) and their 95% CI and calculated in a two-way random effects model based on absolute agreement. The Mann-Whitney test was performed to assess the differences between GoCARB and dietitians’ errors in each of the food categories. Significance was set at the 0.05 level.

## 3. Results

GoCARB and dietitians achieved comparable accuracies in CHO estimation. The mean absolute error of the dietitians in 54 meals was 14.9 (SD 10.12) grams of CHO versus 14.8 (SD 9.73) grams of CHO for the GoCARB system (*p* = 0.93).

**Figure 3:**
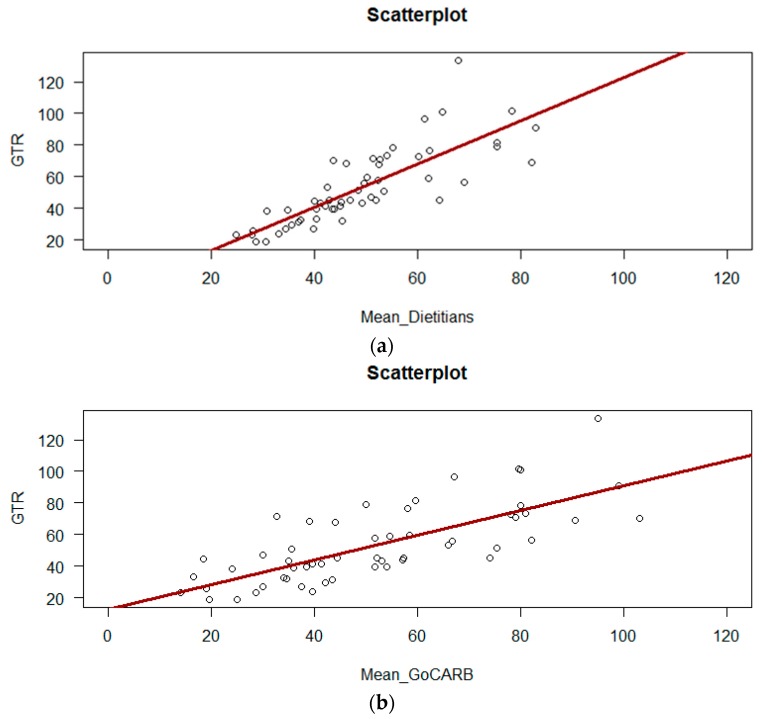
(**a**) Scatterplot of Ground Truth (GTR) and dietitians’ carbohydrates (CHO) estimations (in grams), and (**b**) Scatterplot of Ground Truth (GTR) and GoCARB CHO estimations (in grams).

With regard to the accuracy of the estimations, 35.2% of the dietitians’ CHO estimation error range was within ±10 g, whereas GoCARB achieved 37.03% in the same range. Differences between the visual estimation that was made by dietitians and GTR were significantly and highly correlated (*r* = 0.89, *p* < 0.001), as presented in Figure 3a. The correlation for GoCARB and GTR was also significant (*r* = 0.76, *p* < 0.001), as shown in Figure 3b.

As well as the errors that were made by GoCARB and dietitians per meal, analysis of meal sizes is important. As shown in Table 2, no statistically significant differences were found between the estimations of dietitians and GoCARB for meal size. However, the larger the size of the meal, the greater were the estimation errors in both methods.

**Figure 4:**
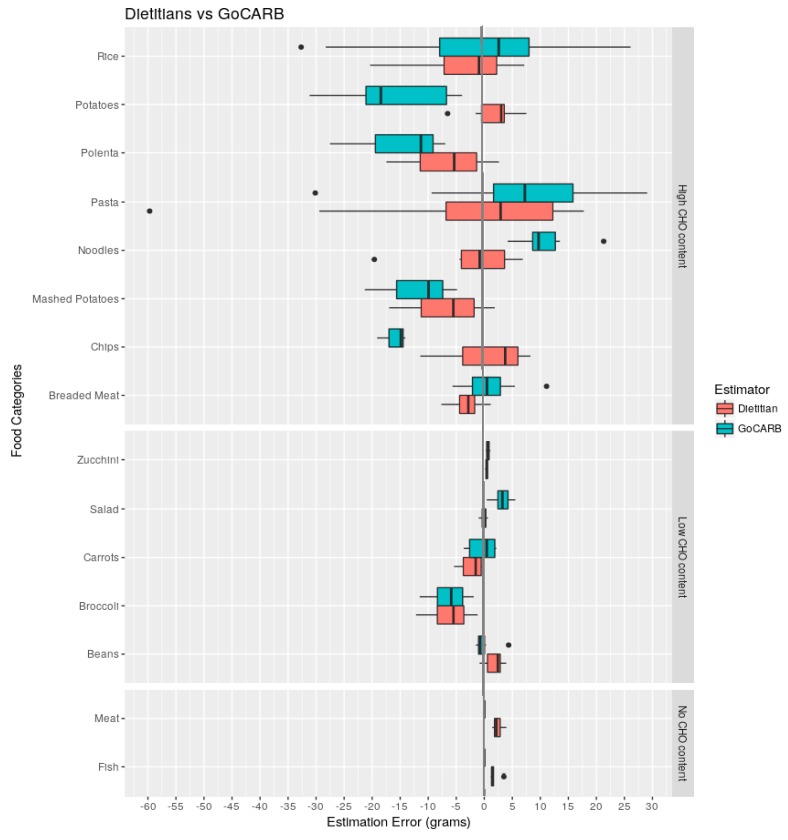
Boxplot of errors per food category for dietitians and GoCARB. Data falling outside the lower and upper quartiles of are plotted as outliers of the data (black points).

The boxplot of Figure 4 illustrates the errors made by the different methods (GoCARB and dietitians) for each food category. The results per food category are as following:

High CHO food categories (breaded meat, chips, mashed potatoes, noodles, pasta, polenta, potatoes, and rice): Both dietitians and GoCARB had difficulty in estimating pasta, rice, and polenta, as demonstrated by the wide range of differences and both over- and under-estimations. GoCARB underestimated potatoes, chips, and mashed potatoes. For mashed potatoes, the same pattern was observed for the dietitians. Dietitians also had a tendency to underestimate the CHO content of breaded meat, while GoCARB overestimated noodles.

Low CHO food categories (beans, broccoli, carrots, salad, and zucchini): Estimation errors made by both methods were small for the zucchini category. While GoCARB overestimates salad, there was a high level of agreement with dietitians, as evidenced in the close to zero median difference and very short whiskers. Dietitians also tended to underestimate carrots. As regards to beans, GoCARB gave good accuracy, but dietitians tended to overestimate. Both methods underestimated broccoli.

No CHO food categories (fish, meat): GoCARB was 100% accurate in estimating fish and meat. On the other hand, dietitians tended to overestimate the CHO content for both fish and meat.

Overall, these results indicate that the higher the CHO content of a food category, the more challenging it is to estimate accurately the amount of CHO content. Moreover, it is observed that GoCARB had considerable difficulty in estimating rice, pasta, potatoes, and mashed potatoes, while dietitians had problems with pasta, chips, rice, and polenta.

Finally, it has to be noted that the result for ICC among dietitians was 0.86 with 95% CI (0.78, 0.91), which implies good to excellent reliability. However, there are differences in the replicability of their estimations, as shown in Table 3.

## 4. Discussion

In this study, we examined the accuracy of the visual estimation method of experienced dietitians in German speaking countries and compared this to the estimations of the GoCARB system. When we compared GTR and visual estimation, we found that both dietitians’ visual estimations and GoCARB’s estimations correlated with the weighing method. Both of the methods had a mean error of less than 15 g. In addition, both dietitians and GoCARB found the CHO content estimation of large meals challenging, with the dietitians reporting higher errors in the cases of pasta, polenta, noodles, salad, and rice. These findings indicate that despite the good performance of both dietitians and GoCARB there is room for improvement, namely in training and more detailed guidelines for dietitians and optimised algorithms for the computational system.

Studies to date have focused on the identification of food items [24] and the estimation of portion size [24,25]. To our knowledge, there is no published study comparing differences in CHO estimation by a computerised system running on smartphone and by dietitians to the actual CHO content of food items. Previous studies that recruited nutrition students and aimed to estimate portion size gave inaccurate results. More specifically, in a study that was conducted in 2010 [26], it was pointed out that only 18.5% of the estimates were considered accurate (±10% of the actual weight). Moreover, another study conducted among nutrition and dietetics students found that only 38% of the estimations reached ±10% of the actual food weight [24]. Thus, it was concluded that nutrition students need more training, in order to quantify the portion size more accurately. Lastly, a comparison study that was implemented between older adults, young adults, and nutritionists regarding portion size estimation, showed that the nutritionists achieved more accurate results when compared to the other groups [27]. In the current study, we compared the visual estimation of dietitians with the GTR and showed that food items, such as pasta, rice, and chips are difficult to estimate, which corroborates previous observations [28]. The results of our study indicate that among vegetables, broccoli, and carrots were mostly underestimated. These results are consistent with those of other studies [24,26] on other cruciferous vegetables, such as cauliflower. In contrast to earlier findings that mixed salads were difficult to estimate [28], the current study found that leafy vegetables were one of the most accurately estimated food category. In another study [29], vegetables tended to be underestimated by direct observation that was made by ten trained individuals. Furthermore, some studies point out that there were significant underestimations in leafy vegetables, and especially lettuce [24,26], while another study conducted in 15 adolescents found out that there was a large overestimation regarding lettuce [30].

There are some possible explanations for the inaccurate estimations that are made by dietitians, which may be attributed to the fact that not all of them used the same method for carbohydrate estimation (four out of six dietitians used the Diabetes Exchange List). Moreover, the lack of specific guidelines for the proposed CHO counting method for breaded food may have contributed to the inaccurate estimation of this food category by dietitians (Figure 1a) [23]. Furthermore, the presence of sauce around the meat as shown in the images may have affected some dietitians’ estimations of meat (Figure 1b), since they added possible CHOs in the sauce on top of their initial estimation. On the contrary, when GoCARB recognises “meat”, the result of zero CHO is automatically recorded. Similarly, there is a thin crust around some images containing fish, and this may have caused dietitians to overestimate fish (Figure 1c). Additionally, a possible explanation for the underestimation of “broccoli” (Figure 1d) from both GoCARB and dietitians might be that “broccoli” belongs to the wide category of “vegetables” in CHO counting guidelines, and thus if it is measured by eye as cups, the corresponding amount of CHO is only 5 g/cup [23]. However, the USDA database (Basic report: 11091, Broccoli, cooked, boiled, drained, without salt) gives 11.20 g of CHO per broccoli cup [31]. The current version of GoCARB needs to be optimised, especially in the food categories of potatoes, chips, polenta, and mashed potatoes, since in these categories there is systemic underestimation, which may influence values for the overall amount of CHO consumed in a meal.

Further research should emphasise on the accuracy of dietitians’ visual estimation of all the macronutrients (CHO, protein, fat), in order to obtain a generalised conclusion on the efficacy of this approach. Moreover, more dietitians should be recruited. Additionally, the food types that are included in the current prototype should be expanded to cover a wider spectrum of eating habits and cultural differences. In this case, GoCARB could be an alternative tool of dietary assessment of daily eating patterns.

The strength of the study is that it is the first study of its kind that compares the visual estimation by expert dietitians with an app. Since the approaches used are standardised, the study can be replicated in different areas in order to give comparable findings. This study has some limitations. Firstly, a small sample of dietitians was included because of the strict inclusion criteria, and especially the fact that the study included only German speaking dietitians, since Central European meals were provided and dietitians from the aforementioned countries were more likely to be familiar with that cuisine. However, this is the primary population on whom we tested GoCARB. Further studies from dietitians of different countries should be carried out, in order to draw a solid conclusion on the visual estimation by dietitians around Europe. Moreover, the meals were not randomly selected, but were organised so that all of them included three different sizes and each of them three different items. As the trial was conducted in a German speaking Swiss hospital and all the meals were prepared from the same kitchen, we cannot assume that all meals with the same ingredients would yield the same result. Furthermore, the estimation of CHO content of the meals may had been more precise, if the dietitians had actually viewed the plates in real time. However, this scenario was not realistic due to dietitians’ residency, as well as the fact that, nowadays, daily practice of the dietitians often includes remote diet assessment of individuals with diabetes based on photo food diaries. Lastly, the meal photos that were provided did not contain overlapping food items. However, the current version of GoCARB and/or dietitians may not perform well in estimating CHO content of dishes containing mixed ingredients (e.g., lasagna), sauces or soups. As mentioned before, the current version of GoCARB contains a limited number of food categories and thus their number should be expanded. Moreover, complex meals are challenging, while other factors that influence blood glucose response, such as glycemic index or presence of fat in the meal, are difficult to be quantified by the used technology, and thus have not been taken into consideration.

In addition, since various platforms and databases receive feedback on meal portion size/nutrient content from different annotators/coders (e.g., healthcare professionals, such as nurses, dietitians or people with unknown profession), there is ambivalence about their qualification, training, and expertise in the field. Since there is lack of information about reliable apps and also insufficient evidence regarding the benefits of each app, it is important that there is an app that performs equivalently to professional dietitians who are experts in the field of carbohydrate estimation. Thus, GoCARB is a decision support system for CHO estimation, which may be beneficial for individuals who cannot accurately estimate the CHO content of the meals and also for those who do not have adequate training in CHO counting. It can also be a useful tool for CHO estimation training of people who have prediabetes and can potentially assist diet assessment of the general population who does not receive relative training. In this way, users and health professionals can receive accurate information and recommend a validated mHealth app to patients.

## 5. Conclusions

This study has investigated possible differences between visual estimation by expert dietitians and the estimations of a computer vision based app, GoCARB. The app may not only make it easier for individuals with diabetes to estimate CHO (by reducing the CHO estimation error), but also offers the option of an easy, accurate, and almost real-time estimation of the CHO content of meals on plates, and thus help to enhance and improve diabetes self-management. GoCARB may also be a useful training tool for those who have limited or no access to dietary education.

## Figures and Tables

**Table 1:** Demographic characteristics of the involved dietitians.

Study Demographics	Dietitians (*n* = 6)
Age (mean, SD)	38.7 years (10.8)
Years of experience	12.2 (9)
Exhibiting T1D	1/6

**Table 2:** Mean (±SD) absolute errors (in grams) of dietitians and GoCARB with respect to meal size.

Meal Size	Absolute Error (grams), Mean ± SD		*p*-Value
	Dietitians	GoCARB	
Small (*n* = 16)	5.9 ± 3.5	8.5 ± 5.6	0.18
Medium (*n* = 16)	7.6 ± 6.3	11.3 ± 8.9	0.27
Large (*n* = 16)	19.4 ± 15.2	20.7 ± 11.6	0.41

**Table 3:** Standard deviation (grams) of replicated results of dietitians’ estimations.

Meal Size	SD (grams) of Dietitians Errors					
	1	2	3	4	5	6
Small (*n* = 14)	3.8	4.7	3.0	7.9	3.6	0.1
Medium (*n* = 14)	4.8	7.5	3.4	5.7	3.7	1.7
Large (*n* = 14)	5.3	6.9	3.5	8.6	6.1	2.7

## Abbreviations

The following abbreviations are used in this manuscript:

T1D	Type 1 diabetes
App	Application
CHO	Carbohydrate
GTR	Ground truth
ICC	Intraclass correlation coefficients

## References

[1] IDF (2015). IDF Diabetes Atlas.

[2] American Diabetes Association (ADA) Standard of Medical Care in Diabetes—2017 (s4–s5, s33–s43). https://professional.diabetes.org/sites/professional.diabetes.org/files/media/standardofcare2017fulldeckfinal_0.pdf.

[3] Fu S., Li L., Deng S., Zan L., Liu Z. (2016). Effectiveness of advanced carbohydrate counting in type 1 diabetes mellitus: A systematic review and meta-analysis. Sci. Rep..

[4] Bell K.J., Barclay A.W., Petocz P., Colagiuri S., Brand-Miller J.C. (2014). Efficacy of carbohydrate counting in type 1 diabetes: A systematic review and meta-analysis. Lancet Diabet. Endocrinol..

[5] Brazeau A.S., Mircescu H., Desjardins K., Leroux C., Strychar I., Ekoé J.M., Rabasa-Lhoret R. (2013). Carbohydrate counting accuracy and blood glucose variability in adults with type 1 diabetes. Diabet. Res. Clin. Pract..

[6] Meade L.T., Rushton W.E. (2016). Accuracy of carbohydrate counting in adults. Clin. Diabet..

[7] Kawamura T., Takamura C., Hirose M., Hashimoto T., Higashide T., Kashihara Y., Hashimura K., Shintaku H. (2015). The factors affecting on estimation of carbohydrate content of meals in carbohydrate counting. Clin. Pediatr. Endocrinol..

[8] Bishop F.K., Maahs D.M., Spiegel G., Owen D., Klingensmith G.J., Bortsov A., Thomas J., Mayer-Davis E.J. (2009). The carbohydrate counting in adolescents with type 1 diabetes (CCAT) study. Diabet. Spectr..

[9] Smart C.E., Ross K., Edge J.A., King B.R., McElduff P., Collins C.E. (2010). Can children with type 1 diabetes and their caregivers estimate the carbohydrate content of meals and snacks?. Diabet. Med..

[10] Smart C.E., King B.R., Mcelduff P., Collins C.E. (2012). In children using intensive insulin therapy, a 20-g variation in carbohydrate amount significantly impacts on postprandial glycaemia. Diabet. Med..

[11] El-Gayar O., Timsina P., Nawar N., Eid W., Elizabeth S. (2013). Mobile applications for diabetes self-management: Status and potential. J. Diabet. Sci. Technol..

[12] Kawano Y., Yanai K. (2015). FoodCam: A real-time food recognition system on a smartphone. Multimed. Tools Appl..

[13] Kong F., Tan J. (2012). DietCam: Automatic dietary assessment with mobile camera phones. Pervasive Mob. Comput..

[14] Puri M., Zhu Z., Yu Q., Divakaran A., Sawhney H. Recognition and volume estimation of food intake using a mobile device. Proceedings of the 2009 IEEE Workshop on Applications of Computer Vision (WACV).

[15] Zhu F., Bosch M., Khanna N., Boushey C.J., Delp E.J. (2015). Multiple Hypotheses Image Segmentation and Classification with Application to Dietary Assessment. IEEE J. Biomed. Health Inform..

[16] Ciocca G., Napoletano P., Schettini R. Food Recognition and Leftover Estimation for Daily Diet Monitoring. Proceedings of the ICIAP 2015 18th International Conference on Image Analysis and Processing.

[17] Anthimopoulos M., Dehais J., Shevchik S., Ransford B.H., Duke D., Diem P., Mougiakakou S. (2015). Computer vision-based carbohydrate estimation for type 1 patients with diabetes using smartphones. J. Diabet. Sci. Technol..

[18] Kitamura K., Yamasaki T., Aizawa K. FoodLog: Capture, Analysis and Retrieval of Personal Food Images via Web. Proceedings of the ACM Multimedia 2009 Workshop on Multimedia for Cooking and Eating Activities.

[19] Beijbom O., Joshi N., Morris D., Saponas S., Khullar S. Menu-match: Restaurant-specific food logging from images. Proceedings of the 2015 IEEE Winter Conference on Applications of Computer Vision, WACV.

[20] Rhyner D., Loher H., Dehais J., Anthimopoulos M., Shevchik S., Botwey R.H., Duke D., Stettler C., Diem P., Mougiakakou S. (2016). Carbohydrate estimation by a mobile phone-based system versus self-estimations of individuals with type 1 diabetes mellitus: A comparative study. J. Med. Internet Res..

[21] Bally L., Dehais J., Nakas C.T., Anthimopoulos M., Laimer M., Rhyner D., Rosenberg G., Zueger T., Diem P., Mougiakakou S. (2017). Carbohydrate estimation supported by the GoCARB system in individuals with type 1 diabetes: A randomized prospective pilot study. Diabet. Care.

[22] Dehais J., Anthimopoulos M., Shevchik S., Mougiakakou S. (2017). Two-view 3D reconstruction for food volume estimation. IEEE Trans. Multimed..

[23] Wheeler M.L., Daly A., Evert A., Franz M.J., Geil P., Holzmeister L.A., Kulkarni K., Loghmani E., Ross T.A., Woolf P. (2008). Choose Your Foods: Exchange Lists for Diabetes, Sixth Edition, 2008: Description and Guidelines for Use. J. Am. Diet. Assoc..

[24] Howes E., Boushey C.J., Kerr D.A., Tomayko E.J., Cluskey M. (2017). Image-based dietary assessment ability of dietetics students and interns. Nutrients.

[25] Williamson D., Allen H., Martin P., Alfonso A., Gerald B., Hunt A. (2003). Comparison of digital photography to weight visual estimation of portion sizes. J. Am. Diet. Assoc..

[26] Japur C.C., Diez-Garcia R.W. (2010). Food energy content influences food portion size estimation by nutrition students. J. Hum. Nutr. Diet..

[27] Timon C.M., Cooper S.E., Barker M.E., Astell A.J., Adlam T., Hwang F., Williams E.A. (2018). A comparison of food portion size estimation by older adults, young adults and nutritionists. J. Nutr. Health Aging.

[28] Byrd-Bredbenner C., Schwartz J. (2004). The effect of practical portion size measurement aids on the accuracy of portion size estimates made by young adults. J. Hum. Nutr. Diet..

[29] Gittelsohn J., Shankar A.V., Pokhrel R.P., West K.P. (1994). Accuracy of estimating food intake by observation. J. Am. Diet. Assoc..

[30] Lee C.D., Chae J., Schap T., Kerr D.A., Delp E.J., Ebert D.S., Boushey C.J. (2012). Comparison of Known Food Weights with Image-Based Portion-Size Automated Estimation and Adolescents’ Self-Reported Portion Size. J. Diabet. Sci. Technol..

[31] US Department of Agriculture, Agricultural Research Service, Nutrient Data Laboratory USDA National Nutrient Database for Standard Reference, Release 28. https://ndb.nal.usda.gov/ndb/.

